# A Diamine Oxidase from *Glutamicibacter halophytocola* for the Degradation of Histamine and Tyramine in Foods

**DOI:** 10.3390/foods14173093

**Published:** 2025-09-03

**Authors:** Lucas Kettner, Alexander Freund, Anna Bechtel, Judit Costa-Catala, Lutz Fischer

**Affiliations:** 1 Department of Biotechnology and Enzyme Science, Institute of Food Science and Biotechnology, University of Hohenheim, Garbenstr. 25, 70599 Stuttgart, Germany; 2 Departament de Nutrició, Ciències de l’Alimentació i Gastronomia, Campus de l’Alimentació de Torribera, Universitat de Barcelona, Av. Prat de la Riba 171, 08921 Santa Coloma de Gramenet, Spain

**Keywords:** diamine oxidase, DAO, histamine, tyramine, histamine intolerance, biogenic amines

## Abstract

A novel diamine oxidase (DAO) was discovered in the bacterium *Glutamicibacter halophytocola* (DAO-GH). The gene of DAO-GH was integrated into the genome of the yeast *Komagataella phaffii* and recombinantly produced under control of the methanol-inducible AOX1 promoter in a bioreactor cultivation. A high DAO activity of 70.2 ± 5.2 µkat/L_culture_ (5.25 ± 0.22 µkat/g_protein_) was yielded after 90 h of cultivation. The DAO-GH was partially purified by the polyethyleneimine precipitation of nucleic acids, fractionated ammonium sulfate precipitation and hydrophobic interaction chromatography, resulting in a specific DAO activity of 19.7 µkat/g_Protein_. The DAO-GH was then biochemically investigated regarding its potential for histamine and tyramine degradation in fermented foods and the human small intestine. Interestingly, the DAO-GH showed activity even at a low pH of 5 and low temperature of 6 °C. Both histamine and tyramine were effectively degraded and DAO-GH showed especially very high affinity towards tyramine (*K*_m_ of 0.009 mM). The DAO-GH was shown to be capable of degrading around 20% of the initially applied histamine in tuna paste (pH 5.6) at 5 °C within 24 h and completely degraded the histamine in a simulated intestinal fluid within 1.5 h in bioconversion experiments. The DAO-GH was spray-dried for the production of a storable enzyme preparation. Only around 17% of activity were lost in this process and the DAO-GH remained stable at room temperature for at least 3 months. The discovery of this DAO with its very advantageous biochemical properties allows the preparation of histamine-reduced or -free fermented foods by a simple enzymatic treatment or the treatment of histamine intolerance symptoms as a dietary supplement or medicine.

## 1. Introduction

Biogenic amines such as histamine and tyramine are low-molecular-weight nitrogenous compounds that are primarily formed by microbial decarboxylation of amino acids during food fermentation [1]. The formation of these compounds in fermented foods is influenced by factors such as the availability of amino acids, the presence of decarboxylase-positive microorganisms, and environmental conditions such as pH, temperature, and salt concentration. Key microorganisms responsible for biogenic amine formation in fermented foods include certain strains of lactic acid bacteria (e.g., Lactobacillus *hilgardii* and *Lactobacillus buchneri*) and certain strains of *Enterobacteriaceae* [1]. Histamine is considered one of the most relevant biogenic amines due to its various physiological functions, and histamine intolerance is gaining increasing recognition in the medical and scientific communities. Histamine intolerance refers to the inability to adequately break down amounts of histamine from food that would not normally cause adverse physiological symptoms in healthy people [1]. Symptoms of this intolerance can be very diverse due to the high physiological importance of histamine as an endogenous compound with its various functions as neurotransmitter and hormone. They range from skin redness and itching, diarrhea, vomiting, and abdominal pain to effects on the cardiovascular system (e.g., hypotension, dizziness, or tachycardia) [2]. Histamine intolerance cannot currently be objectively diagnosed due to this variety of symptoms as well as the lack of diagnostic parameters [2].

Histamine intolerance appears to be due to an impairment of the available activity of histamine-degrading diamine oxidase (DAO; EC 1.4.3.22), which is particularly responsible for the degradation of histamine from food in the small intestine [3]. The DAO belongs to the enzyme class of oxidoreductases and catalyzes the oxidative deamination of primary amines with a preference for diamines [4,5,6]. Besides the lack of an objective diagnosis, histamine intolerance also cannot be currently treated by medication or DAO supplementation [2]. The guidelines on the management of suspected adverse reactions to ingested histamine [2] propose a personalized and symptom-oriented nutritional therapy to prevent symptoms of histamine intolerance. The formation of histamine in foods is mainly due to microbial decarboxylase activity (L-histidine decarboxylase; EC 4.1.1.22); therefore, histamine is found not only in spoiled foods, but more relevantly in fermented foods, such as cheese, sausage or wine [7]. Therefore, the microorganisms in commercial starter cultures should be specifically selected to be decarboxylase-negative and optimally suppress the growth of undesired decarboxylase-producing microorganisms [8]. Some studies have investigated commercial starter cultures, and did not find any considerable potential for biogenic amine formation [9,10]. However, this might not apply to all commercial starters and the contamination with decarboxylase-positive microorganisms and, thus, biogenic amine formation still seems to be an issue. In fact, various biogenic amines can be found in foods. The most common ones are histamine, tyramine, cadaverine, 2-phenylethylamine, spermine, spermidine, putrescine, tryptamine and agmatine [11]. Among the biogenic amines, histamine and tyramine are particularly relevant from a toxicological and food safety point of view [12].

Additionally, the biogenic amine content can be reduced during the fermentation or ripening of foods by the inoculation of amine-degrading starters [13,14]. Furthermore, the direct application of a DAO enzyme preparation has been investigated and discussed recently [15,16,17,18]. Although this approach has advantages, such as a high degree of standardization and the avoidance of microbial-induced off-flavors, there are some limitations that severely restrict the direct use of DAO in foods, especially in fermented foods [17]. This is because the environmental conditions in fermented foods are unfavorable due to a rather acidic pH value and lower temperatures. Consequently, enzyme catalysis can either proceed very slowly or not at all with the current DAOs available [17]. Direct biogenic amine degradation in fermented foods, therefore, requires a DAO that has sufficient activity in the acidic pH range and at lower temperatures (below 20 °C), a high affinity for relevant biogenic amines, such as histamine and tyramine, and, above all, can be produced biotechnologically with a high specific enzyme activity.

This study focused on the investigation of a newly discovered microbial DAO regarding its potential suitability for biogenic amine degradation in fermented foods and in the small intestine.

## 2. Materials and Methods

### 2.1. Materials and Reagents

Yeast extract was obtained from Acros Organics (New Jersey, NJ, USA). Tryptone (enzymatically digested casein), sodium diethyldithiocarbamate and ortho-phosphoric acid (H_3_PO_4_) were obtained from Merck KGaA (Darmstadt, Germany). 1,4-piperazinediethanesulfonic acid (PIPES), MES, TRIS, sodium acetate, histamine dihydrochloride, tyramine dihydrochloride, sodium hydroxide (NaOH), monobasic potassium phosphate (KH_2_PO_4_), di-potassium hydrogen phosphate (K_2_HPO_4_), ammonium sulfate, hydrochloric acid (HCl), hydrogen peroxide (30%), sodium chloride (NaCl), sorbitol, glucose and agar-agar were obtained from Carl Roth GmbH (Karlsruhe, Germany). Peptone (enzymatically digested casein) and polyethyleneimine solution (average M_n_ ~60,000 by GPC, average M_w_ ~750,000 by LS) were obtained from Sigma-Aldrich (Taufkirchen, Germany). Zeocin was obtained from InvivoGen (San Diego, CA, USA). Kanamycin sulfate, chloramphenicol and bovine serum albumin (BSA; modified Cohn Fraction V, pH 5.2) were obtained from Serva electrophoresis GmbH (Heidelberg, Germany). TaKaRa Ex Taq^®^ DNA polymerase was obtained from Takara Bio Inc. (Kusatsu, Japan). The Precision Plus Protein™ unstained protein standard 10–250 kDa was purchased from Bio-Rad laboratories GmbH (Feldkirchen, Germany). (10-(carboxymethyl-aminocarbonyl)-3, 7-bis(dimethylamino) phenothiazine sodium salt (DA-67) was purchased from Fujifilm Wako Chemicals U.S.A. Corp (Richmond, VA, USA). Horseradish peroxidase (Grade I) was purchased from AppliChem GmbH (Darmstadt, Germany).

### 2.2. Strains and Media

*Glutamicibacter halophytocola* was cultivated in a standard culture medium containing 15 g/L peptone, 3 g/L yeast extract, 6 g/L NaCl, 1 g/L glucose, 1 g/L glycerol (pH adjusted to pH 7 with 5 M NaOH).

Plasmid construction and propagation was performed in *Escherichia coli* XL-1, grown in lysogeny broth media with the required antibiotic (30 µg/mL kanamycin or 40 µg/mL chloramphenicol). *Komagataella phaffii* ATCC 76273 was purchased from the American Type Culture Collection (Manassas, VA, USA) and cultivated at 30 °C. *K. phaffii* transformants were grown on YPDS agar plates that contained yeast extract (10 g/L), peptone (20 g/L), glucose (20 g/L), sorbitol (1 M), agar-agar (15 g/L) and zeocin (100 µg/mL) for selection. For cultivation of recombinant *K. phaffii*, the following media were used: YPD medium (10 g yeast extract per L, 20 g peptone per L, 20 g glucose per L), buffered glycerol complex medium (BMGY) or buffered methanol complex medium (BMMY) (10 g yeast extract per L, 20 g peptone per L, 100 mM potassium phosphate buffer (pH 6), 13.4 g yeast nitrogen base per L, 0.4 mg biotin per L, 10 g glycerol per L or 5 mL methanol per L), buffered minimal glycerol medium (100 mM potassium phosphate buffer (pH 5), 13.4 g yeast nitrogen base per L, 0.4 mg biotin per L, 10 glycerol per L) and basal salts minimal medium (26.7 mL phosphoric acid (85%) per L, 0.93 g calcium sulfate per L, 18.2 g potassium sulfate per L, 14.9 g magnesium sulfate-7∙H_2_O per L, 4.13 g potassium hydroxide per L, 40 g glycerol per L at start of batch phase) containing 4.35 mL PTM_1_ trace salts solution per L [19].

### 2.3. Identification of the DAO Gene in Glutamicibacter Halophytocola

In a preliminary investigation, an agar-based screening (as known in the art) revealed the microorganism *Glutamicibacter halophytocola* (identified by 16s rDNA sequencing) to be a DAO producer. The gene coding for the DAO was identified in silico using the BLAST program (https://blast.ncbi.nlm.nih.gov) with the amino acid sequences of the DAOs from *Yarrowia lipolytica* (accession: Q6CGT2) and *Arthrobacter crystallopoietes* (accession: WP_074701741.1) as entries. This gene was amplified by polymerase chain reaction from genomic DNA of *G. halophytocola* using the primers 5′-ATGGAACACCTTCACCCAACGA-3′ and 5′-CTAGGCTCCGCAGTGTCCTTCGGTTTCAGG-3′ and sent for sequencing (Eurofins Genomics GmbH, Ebersberg, Germany).

### 2.4. Construction and Investigation of K. phaffii Clones

The gene of the DAO-GH from *G. halophytocola* was codon-optimized for production in *Komagataella phaffii* and synthesis was carried out by Invitrogen (Thermo Fisher Scientific, Waltham, MA, USA).

The construction of the cassette plasmid containing the DAO-GH expression cassette was performed with the MoClo Yeast and MoClo *Pichia* Toolkit [20,21]. The DAO-GH gene was flanked by the restriction sites *Bsm*BI and *Bsa*I and specific overhangs, as described by Bechtel et al., 2025 [22]. Part plasmids used for the assembly of the cassette plasmid are shown in the Supplementary Material (Table S1). The cassette plasmid map is shown in Figure S1.

The DAO-GH expression cassette plasmid was linearized by *Avr*II and homologous recombinant integration into the genome of *K. phaffii* was performed, as described by Bechtel et al., 2025 [22]. Recombinant *K. phaffii* clones (6 in total) were investigated for their intracellular DAO activity after cultivation in BMGY/BMMY medium in deep well plates (96/2000 µL; Eppendorf AG, Hamburg, Germany), as described by Bechtel et al., 2025 [22].

### 2.5. Bioreactor Cultivation of Recombinant K. phaffii for the Production of DAO-GH

The recombinant *K. phaffii* strain with the highest intracellular DAO-GH activity was used for a fed-batch bioreactor cultivation in Multifors 2 bioreactors (1.4 L vessel volume; Infors HT, Bottmingen, Switzerland), according to Bechtel et al., 2024 [23], with slight modifications. The depletion of glycerol was not verified quantitatively but rather based on an increase in the pO_2_ signal. The methanol feed rates were also modified. After 21.6 h of batch cultivation on glycerol, the methanol feed was started and a feed rate of 1.8 mL/h was used for 12 h. This was followed by feed rates of 3.6 mL/h for 8.2 h, 5.4 mL/h for 2.7 h, 7.2 mL/h for 5 h, 9 mL/h for 3.2 h and 10.8 mL/h for 42.6 h. The absence of an accumulation of methanol was regularly qualitatively verified by pausing the methanol feed and observing the pO_2_ signal. In the case of an immediate increase in the pO_2_ signal, it was concluded that no methanol was accumulated and the feed rate was either maintained or further increased. If necessary, antifoam 204 (Sigma-Aldrich, St. Louis, MO, USA) was added to counteract any foam formation. Samples of 5 mL were taken regularly during the cultivation and centrifuged (13,000× *g*, 4 °C, 5 min). Washing of the cell pellets was performed with saline and washed cell pellets were subsequently stored at −20 °C until cell disruption. Additionally, samples of 1 mL were regularly taken to determine the optical density and bio dry mass. At the end of the bioreactor cultivation, the remaining cells were harvested by centrifugation (6000× *g*, 4 °C, 15 min), washed with saline and again centrifuged (8000× *g*, 4 °C, 15 min). The cell pellet was stored at −20 °C until further use.

### 2.6. Disruption of K. phaffii Cells

*K. phaffii* cells obtained from the deep well plate cultivation for clone selection were suspended in PIPES buffer (25 mM, pH 7.2) and disrupted in microtiter plates, according to Bechtel et al., 2024 [23].

Cells obtained over the course of the bioreactor cultivation were suspended (30% (*w*/*v*)) in PIPES buffer (25 mM, pH 7.2) and disrupted in 2 mL Eppendorf reaction tubes using the TissueLyser II (Qiagen, Hilden, Germany) at 30 Hz for 30 min. The supernatant obtained after centrifugation (13,000× *g*, 4 °C, 5 min) was directly used for the investigation of its protein content and DAO activity. 226.3 g *K. phaffii* bio wet mass was used to prepare a 30% (*w*/*v*) suspension. Cell disruption was performed with the DYNO^®^-MILL KDL A (Willy A. Bachofen GmbH, Nidderau, Germany) using glass beads (0.75 mm diameter), according to Bechtel et al., 2024 [23], for the purification of DAO-GH. After centrifugation (16,266× *g*, 4 °C, 45 min), DAO-GH was further purified.

### 2.7. Partial Purification of DAO-GH

The DAO-GH was partially purified by the precipitation of nucleic acids using polyethyleneimine (PEI), fractionated ammonium sulfate precipitation and hydrophobic interaction chromatography (HIC), according to Bechtel et al., 2025 [22], using minor modifications. All centrifugation steps were conducted at 16,266× *g* and 4 °C for 45 min. After PEI precipitation, the fractionated ammonium sulfate precipitation was performed by increasing the ammonium sulfate saturation to 25% to remove foreign proteins and then to 60% to finally precipitate the DAO-GH. The DAO-GH pellet obtained was dissolved in binding buffer (25 mM sodium phosphate, pH 7.0, containing 1.3 M (NH_4_)_2_SO_4_) in a final volume of 670 mL and purified by HIC using the column material Toyopearl Phenyl-650 M (Tosoh Bioscience, Tokyo, Japan) (CV = 350 mL). The purification protocol was performed at a constant flow rate of 30 mL/min. After the sample application, non-bound proteins were washed out with binding buffer over 3 CV. The concentration of ammonium sulfate was then reduced to 50% over 4 CV. This concentration was held for 3 CV and DAO-GH was eluted by decreasing the ammonium sulfate concentration to 0% over 0.5 CV. This step was held for 2.5 CV. The DAO-containing fraction was investigated regarding the DAO activity and protein concentration and was qualitatively analyzed by sodium dodecyl sulfate-polyacrylamide gel electrophoresis (SDS-PAGE).

### 2.8. Protein Analysis

The protein content of enzyme samples was determined, according to Bradford [24], using BSA as a standard. Samples of the DAO purification procedure were analyzed by SDS-PAGE on a 10% separating gel [25]. An amount of 5 µg protein was loaded onto each lane of the SDS-PAGE. A protein molecular mass standard was used (Precision Plus Protein™ unstained protein standard 10–250 kDa) for the molecular mass estimation. Coomassie Brilliant Blue R-250 was used to stain the gel [26]. The native molecular weight of DAO-GH was determined by size exclusion chromatography (SEC) using the HiLoad 16/600 Superdex 200 prep grade (Cytiva, Marlborough, MA, USA) column on the Äkta pure chromatography system (Cytiva, USA). The HIC-purified DAO-GH was desalted against SEC buffer (50 mM sodium phosphate buffer and 150 mM NaCl; pH 7) using PD MiniTrap desalting columns with Sephadex G-25 resin, according to the manufacturer’s instructions (Cytiva, Marlborough, MA, USA). An amount of 1 mL of this DAO-GH sample was then loaded into the SEC column. The chromatographic separation was performed at a constant flow rate of 1 mL/min and with SEC buffer. Fractions were collected and analyzed for DAO activity. The gel filtration calibration kit (Cytiva, USA) for high molecular weight was used for the determination of the native molecular weight, and separated as described for the DAO-GH sample.

### 2.9. DAO Activity Determination

The DAO-GH activity was determined using DA-67 assay [27]. The reaction mixture contained the following components: 375 µL histamine solution (2.79 mM; dissolved in 25 mM potassium phosphate buffer; pH 6.8) and 363 µL DA-67 reagent (10-(carboxymethyl-aminocarbonyl)-3, 7-bis(dimethylamino) phenothiazine sodium salt; 50 µM; dissolved in 25 mM potassium phosphate buffer; pH 6.8) and was incubated at 37 °C for 10 min and stirred at 850 rpm. Subsequently, 12 µL (266 units·mL^−1^) of horseradish peroxidase (Grade I) (Carl Roth GmbH + Co. KG, Karlsruhe, Germany) were added. 25 µL DAO solution were added to start the reaction. The reaction was then incubated at 37 °C and 850 rpm. 25 µL sodium diethyldithiocarbamate (30 mM) were added to stop the reaction. The absorption was measured at 620 nm. The histamine solution was replaced with buffer (25 mM potassium phosphate buffer; pH 6.8) for reference. Hydrogen peroxide (0.5–10 nmol·mL^−1^) was used for the calibration. The enzyme activity was calculated in nkat, whereby 1 nkat converts 1 nmol substrate·s^−1^ at 37 °C.

### 2.10. Investigation of the Temperature and pH Profile of DAO-GH

The influence of temperature on the enzyme activity of DAO-GH was investigated under standard assay conditions, whereby the incubation temperature was varied between 6 and 70 °C. The pH-dependency of the DAO-GH activity was investigated under standard assay conditions at 37 °C, whereby the following buffer systems were used (each at 50 mM): sodium acetate buffer (pH 4.5, 5.0, 5.5), MES buffer (pH 5.5, 6.0), potassium phosphate buffer (pH 6.0, 6.5, 6.8), PIPES buffer (pH 6.8, 7.0, 7.5) and TRIS buffer (pH 7.5, 8.0, 8.5, 9.0). The respective buffer systems were used for separate calibrations with hydrogen peroxide (0.5–10 nmol/mL).

### 2.11. Kinetic Characterization of DAO-GH

The apparent kinetic parameters of DAO-GH were determined by Michaelis-Menten kinetics with histamine and tyramine as the substrates under standard assay conditions. The histamine concentration was varied between 0.05 and 3.1 mM and the tyramine concentration between 0.005 and 3 mM. Kinetic investigations were performed within the initial reaction velocity.

Besides histamine and tyramine, the DAO-GH activity was also determined with the biogenic amines putrescine and cadaverine at concentrations of 0.1, 1 and 5 mM.

### 2.12. Stability of DAO-GH in Simulated Intestinal Fluid (SIF)

The stability of DAO-GH was tested in SIF with and without the addition of established food constituents to simulate possible food matrices, according to Kettner et al., 2022 [28]. Therefore, SIF (containing pancreatin) was prepared in accordance with the United States Pharmacopeia [29]. The SIF was supplemented with BSA, whey protein isolate and sodium caseinate, each at 16.67 g/L, and sucrose at 50 g/L, for the approach simulating a possible food matrix. The DAO-GH was incubated in the pure SIF and the SIF supplemented with food matrix at 37 °C. Samples of 100 µL were taken over the course of incubation and were directly used for DAO activity determination using the DA-67 assay.

### 2.13. Spray-Drying of DAO-GH

The DAO-GH was spray-dried using maltodextrin as a carrier material for the production of a storable DAO preparation. Thus, a 20% (*w*/*v*) maltodextrin (dextrose equivalent 16–19) solution was prepared by dissolving maltodextrin in sodium phosphate buffer (25 mM; pH 7.0) and adjusting the pH to 7.0 with 4 M NaOH. The 20% (*w*/*v*) maltodextrin solution was then mixed in a 1:1 ratio with the partially purified DAO-GH resulting in a total volume of 104 mL. Spray drying was performed using the Büchi Mini Spray Dryer B-290 (BÜCHI Labortechnik GmbH, Essen, Germany) with a constant outlet temperature of 90 °C (inlet temperature 160 °C, aspirator 35 m^3^/h, pump rate 6 mL/min, spray rate 667 L/h, nozzle cap diameter 1.5 mm). The residual DAO activity was determined as well as the water activity value using the HygroPalm (Rotronic Messgeräte GmbH, Ettlingen, Germany).

### 2.14. Degradation of Histamine in a Tuna Paste

Tuna was purchased from a local supermarket (tuna filet in its own juice; from EDEKA, Stuttgart, Germany) and was spiked with histamine at 150 mg/kg. Tap water was added to 100 g of tuna to prepare a 40% (*w*/*v*) suspension and then homogenized using a blender. The DAO-GH powder was added to the suspension at 0.27% (*w*/*v*) (equal to 40 nkat) and was mixed using a blender again. The tuna paste was divided in three equal parts and incubated in 50 mL falcon tubes at 5 °C for 24 h. Samples of 5 mL were taken and immediately inactivated in a water bath at 95 °C for 10 min before and after this incubation. Subsequently, the samples were centrifuged (20,000× *g*, 4 °C, 10 min) and the supernatants were used to determine the histamine concentration by RP-UHPLC.

### 2.15. Degradation of Histamine in SIF

A bioconversion of histamine (150 mg/kg; 1.35 mM) was performed in SIF in the presence of established food constituents, according to Kettner et al., 2022 [28], but in a 5 mL scale. The DAO-GH powder (2.6 g/L; 2400 nkat/L; activity determined by the RP-HPLC analysis of the imidazole-4-acetaldehyde formation, as described by Kettner et al., 2020 [30]), was added to the bioconversion experiment and the reaction was incubated at 280 rpm and 37 °C for 90 min. Samples of 1 mL were taken at the beginning and after 90 min, and immediately inactivated in a water bath at 95 °C for 10 min. The samples were centrifuged (20,000× *g*, 4 °C, 10 min) and the supernatants were analyzed for their histamine concentration by RP-UHPLC.

### 2.16. UHPLC-FL Determination of Histamine

The histamine concentration was determined in tuna paste and SIF by UHPLC-FL (ultra high-performance liquid chromatography; fluorescence detector), according to Latorre-Moratalla et al., 2009 [31]. The Waters Acquity™_._ Ultra Performance Liquid Chromatography apparatus equipped with a quaternary pump, an autosampler and a fluorescence detector were used and histamine was separated on an Acquity UPLC BEH C18 column (1.7 µm, 2.1 × 50 mm) (Waters Corp., Milford, MA, USA), which was kept in an oven at 42 °C.

Histamine was derivatized post-column with *o*-phthalaldehyde (0.01% (*w*/*v*)) and detected by subsequent fluorescence detection (λ_ex_: 340 nm and λ_em_: 445 nm). The mobile phase was pumped at a flow rate of 0.8 mL/min, while the derivatization reagent was pumped at 0.4 mL/min. The mobile phase and OPA derivatization reagent were prepared as described by Latorre-Moratalla et al., 2009 [31]. The injection volume was set to 1 µL for both the standard and sample solutions. Data acquisition and processing was performed with the software Empower™ 3 (Waters Corp., Milford, MA, USA).

### 2.17. Statistical Analysis

All experiments were executed at least in duplicate and evaluated by determining the standard deviation with Excel (Microsoft, Redmond, VA, USA). Data are presented as mean values with standard deviation. Enzyme kinetics were evaluated by nonlinear regression using the data analyzing software Sigmaplot 12.5 (Systat Software GmbH, Erkrath, Germany).

## 3. Results and Discussion

### 3.1. Discovery of the New Diamine Oxidase (DAO-GH) in Glutamicibacter Halophytocola

In a preliminary investigation, an agar-based screening for DAO-producing microorganisms was performed and *Glutamicibacter halophytocola* was found and identified. The gene coding for a putative primary amine oxidase (accession: WP_060702819.1) in *G. halophytocola* was identified using the BLAST program (https://blast.ncbi.nlm.nih.gov) with the amino acid sequences of the DAOs from *Yarrowia lipolytica* (accession: Q6CGT2) and *Arthrobacter crystallopoietes* (accession: WP_074701741.1) as entries. Comparing the amino acid sequences of the DAOs from *Y. lipolytica* and *A. crystallopoietes*, the putative primary amine oxidase from *G. halophytocola* showed a percent identity of 39% (query cover 64%) and 62% (query cover 97%), respectively.

Sadeghi et al., 2020 [32] isolated the histamine-degrading bacterium *Glutamicibacter* sp. N1A3101 from a soil sample obtained around the roots of stinging nettle (*Urtica dioica*) in Iran. The authors provided a partial nucleotide sequence of the DAO (accession: MT993978). Aligning this partial nucleotide sequence with the nucleotide sequence of the DAO-GH showed no significant similarities.

The *G. halophytocola* from this work was cultivated in a shake flask (in standard culture medium) to investigate its native DAO activity. After around 6 h of cultivation, an optical density (OD_600_) of 0.8 and an intracellular DAO activity of 0.65 nkat/L_culture_ was determined (no data shown). After around 25 h of cultivation, the level of native DAO activity decreased to approximately 0.12 nkat/L culture. However, as native DAO production was not the focus of this study, it was not investigated further.

The gene coding for the putative primary amine oxidase in *G. halophytocola* was amplified from its genome and used for the heterologous recombinant expression in *Escherichia coli* BL21 (DE3) and *Bacillus subtilis* SCK6 in preliminary experiments (no data shown). The recombinant expression proved that the putative “primary amine oxidase” is an active DAO. However, low activity yields of 3 nkat/L_Culture_ (0.01 nkat/mg_Protein_) in *E. coli* and 64 nkat/L_Culture_ (0.23 nkat/mg_Protein_) in *B. subtilis* were found. The nucleotide and amino acid sequences of the DAO-GH are shown in Figure S2A,B.

### 3.2. Production of the DAO-GH in Komagataella phaffii

Heterologous DAO-GH production was carried out with *K. phaffii* using the methanol-inducible AOX1 promoter (pAOX1). The integration of the cassette plasmid into the genome of *K. phaffii* was verified by polymerase chain reaction (Figure S3). Initially, the recombinant *K. phaffii* clones were cultivated in deep well plate cultivations for investigation of the intracellular DAO-GH activity (Figure S4). Clone 1 showed the highest DAO-GH activity (1.86 ± 0.02 µkat/L_culture_) in this deep well plate screening and was used for all further experiments.

**Figure 1:**
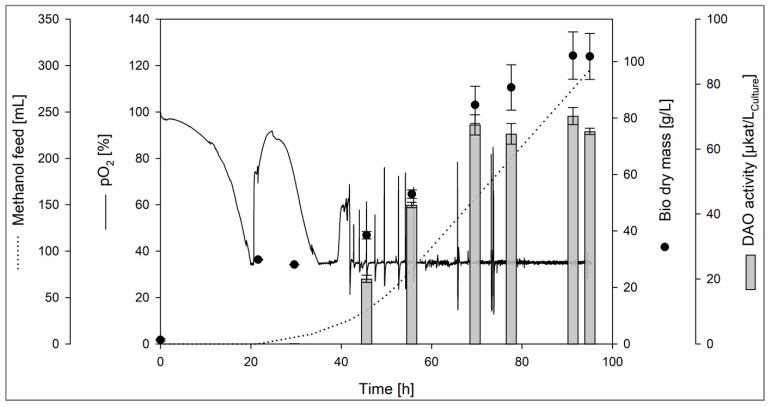
Bioreactor cultivation (fed-batch) of the recombinant *K. phaffii* for the DAO-GH production. Basal salts minimal medium [carbon sources: glycerol (batch) and methanol (fed-batch)], 30 °C, pH 5.

The recombinant *K. phaffii* clone was used for DAO-GH production in a fed-batch bioreactor cultivation. Here, the recombinant *K. phaffii* was first grown on glycerol as the carbon source to generate biomass. After the glycerol has been depleted (indicated by an increase in pO_2_), the methanol feed was started, inducing the DAO-GH expression. The methanol feed was gradually increased and stopped or reduced whenever methanol accumulated (Figure 1).

The maximum DAO activity of 70.2 ± 5.2 µkat/L_culture_ (5.25 ± 0.22 µkat/g_protein_) was found after 90 h of cultivation. The bio dry mass at this time point was around 102 g/L. This resulted in a specific DAO activity per gram bio dry mass of 0.69 ± 0.08 µkat/g. The volumetric DAO activity was around 40-fold higher than the activity observed in the deep well plate screening.

By comparison, the recombinant production of another DAO, the DAO-1 from *Yarrowia lipolytica*, in *K. phaffii* yielded a volumetric DAO activity of around 230 µkat/L_culture_ [22]. However, the activity of the DAO-1 was determined under optimal conditions for maximal DAO-1 activity (pH 7.2, 37 °C, 14.52 mM histamine), whereas the activity of the DAO-GH in this study was investigated under conditions that were chosen in prior studies as physiologically relevant conditions (pH 6.8, 37 °C, 1.35 mM histamine) [28,30,33]. It has to be considered that the activity of the DAO-1 from *Y. lipolytica* is about 50% lower under these conditions.

### 3.3. Partial Purification of DAO-GH

The DAO-GH was purified by the polyethyleneimine precipitation of nucleic acids, fractionated ammonium sulfate precipitation and HIC. 33 µkat of DAO-GH activity (specific DAO-GH activity of 3.2 µkat/g_protein_) were obtained in the crude extract from the disruption of 226.3 g of *K. phaffii* bio wet mass.

The partial purification of the DAO-GH by HIC (Figure S5) yielded a total of 15.7 ± 0.4 µkat (48% yield) with a specific DAO-GH activity of 19.7 µkat/g_Protein_ (purification factor of around 6) (Table 1).

**Figure 2:**
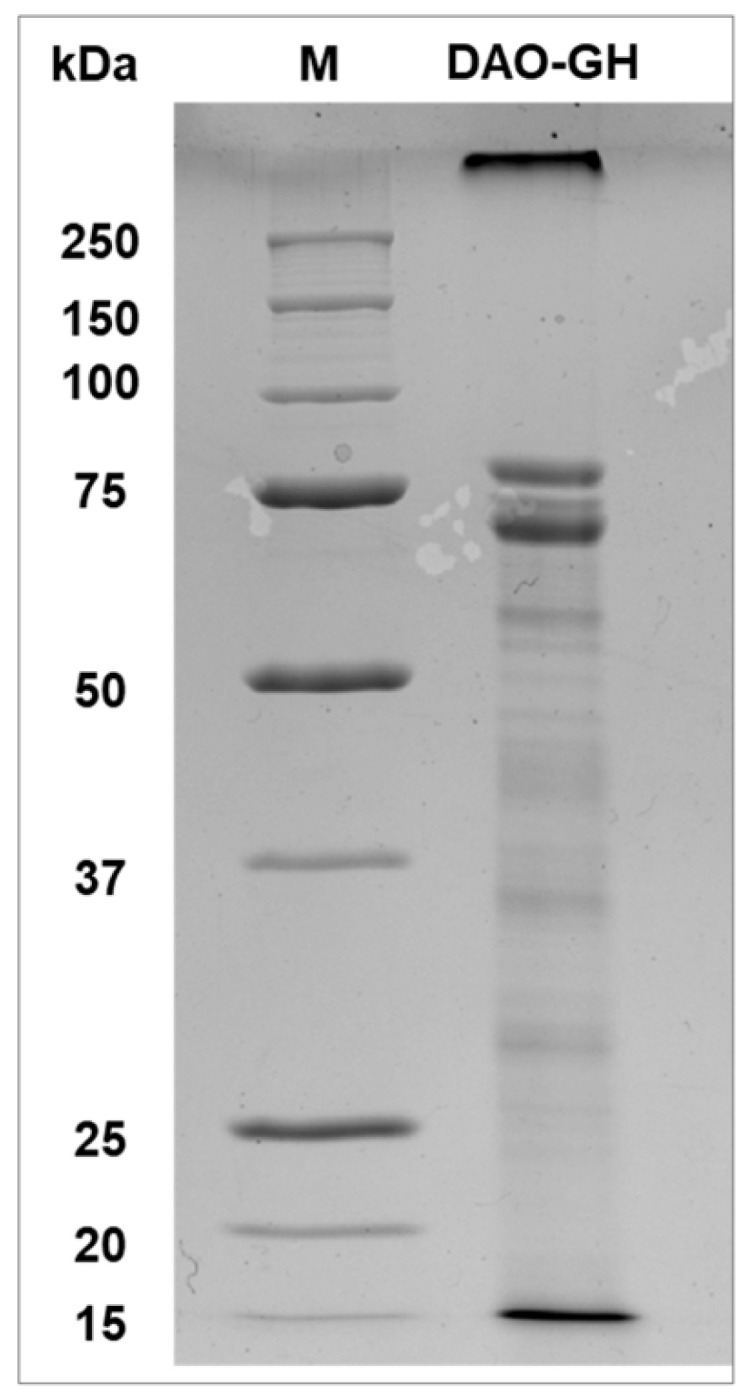
SDS-PAGE analysis of the partially purified DAO-GH (10% (*w*/*v*) acrylamide gel). Staining was performed using Coomassie brilliant blue R-250. An amount of 5 µg protein of DAO-GH was loaded on the gel. M = Precision Plus Protein™ unstained protein standard 10–250 kDa.

The partially purified DAO-GH was investigated by SDS-PAGE and showed two distinct protein bands at around 75 kDa (Figure 2). Although the theoretical molecular weight of the DAO-GH is 72.48 kDa (calculated from the amino acid sequence), MS-analysis revealed that the band slightly above 75 kDa was indeed the DAO-GH (internal communication). The protein band slightly below 75 kDa might be the alcohol oxidase 1 (AOX1; Uniprot ID: P04842), which has a theoretical molecular weight of around 74 kDa.

The native molecular weight of DAO-GH was determined by SEC and was found to be around 154.5 kDa (Figure S6). Thus, concluding from the molecular weight of its monomer and the experimentally determined native molecular weight, the DAO-GH seems to be a homodimeric protein.

### 3.4. Biochemical Investigation of DAO-GH

**Figure 3:**
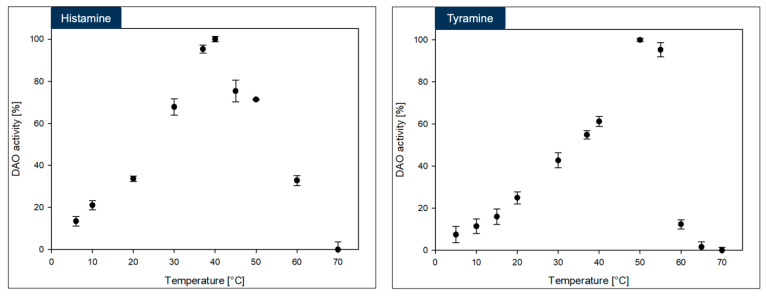
Influence of temperature on the DAO-GH activity with histamine and tyramine as substrates. The DAO-GH activity was determined in potassium phosphate buffer (25 mM, pH 6.8). 100% DAO activity = 6.7 ± 0.09 µkat_Histamine_/g_Protein_ and 12.49 ± 0.09 µkat_Tyramine_/g_Protein_.

**Figure 4:**
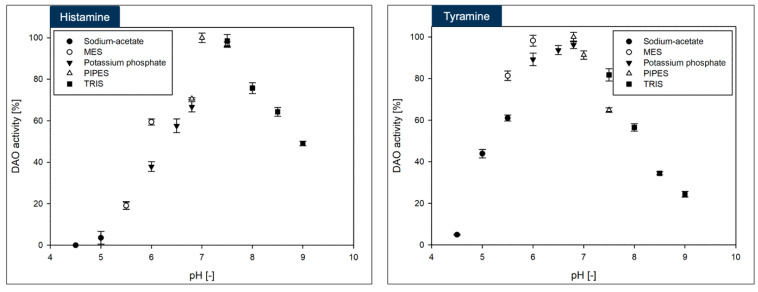
Influence of pH-value and buffer (each at 50 mM) on the DAO-GH activity with histamine and tyramine as substrates. The DAO-GH activity was determined at 37 °C. 100% DAO activity = 9.4 ± 0.2 µkat_Histamine_/g_Protein_ and 10.75 ± 0.25 µkat_Tyramine_/g_Protein_.

The DAO-GH showed rather broad temperature and pH profiles when compared to other DAOs [17] (Figure 3 and Figure 4). The maximum DAO-GH activity was at 40 °C and between pH 7.0 and 7.5 (PIPES buffer) with histamine and at 50 °C and between pH 5.5 and 7.5 (PIPES buffer) with tyramine as substrates. The higher temperature maximum with tyramine was attributed to a potentially higher thermal stability of DAO-GH in the presence of this substrate. Interestingly, the DAO-GH still showed around 13% of its maximal activity with histamine at 6 °C. By comparison, a recently published DAO (DAO-1) from *Y. lipolytica* showed only around 10% of its maximal activity at 20 °C [33]. Moreover, the DAO-GH showed activity at a neutral to slightly acidic pH-value, which is desirable for the application in fermented foods [17]. The DAO-GH still showed around 4 and 20% of its activity towards histamine even at a pH of 5.0 and 5.5, respectively. The DAO-GH with tyramine still showed activity of around 5% of its maximal activity at pH 4.5 and around 45% at pH 5. By comparison, the DAO-1 from *Y. lipolytica* [33], a phenylethylamine oxidase from *Arthrobacter globiformis* [34] and a monoamine oxidase from *Mycobacterium* sp. strain JC1 [35] seemed to show weak or even no activity in this pH range. The DAOs seem to generally show maximal activity in the neutral pH range and tend to have low or no activity under acidic conditions [17].

In conclusion, the DAO-GH might be applicable for the histamine and tyramine degradation in fermented foods which exhibit an acidic pH and at temperatures commonly found in fermentation processes [17].

The maximum DAO-GH activity was at around pH 7.0 and sufficient activity was present at 37 °C. Hence, the DAO-GH could also be orally administered to degrade dietary biogenic amines, such as histamine and tyramine in the human small intestine [28].

### 3.5. Kinetic Investigation of DAO-GH

**Figure 5:**
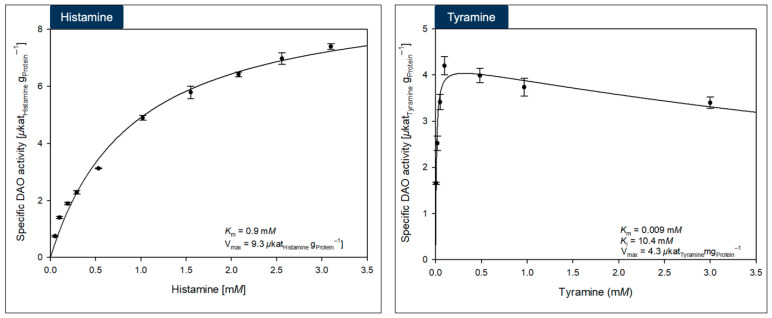
Michaelis-Menten kinetics of the DAO-GH with histamine and tyramine as substrates. The DAO-GH activity was determined in potassium phosphate buffer (25 mM, pH 6.8) at 37 °C.

The DAO-GH was investigated regarding its affinity to the substrates histamine and tyramine (Figure 5) (Michaelis-Menten kinetics).

The *K*_m_ of the DAO-GH with histamine as substrate was 0.9 ± 0.07 mM (V_max_ = 9.3 ± 0.27 µkat_Histamine_/g_Protein_; R^2^ = 0.986). A further increase in the histamine concentration to 6 mM did not lead to a further increase or decrease in DAO-GH activity, proving that DAO-GH is not substrate-inhibited by histamine. By contrast, the DAO-1 from *Y. lipolytica* showed a higher *K*_m_ of 2.3 mM with histamine and was substrate-inhibited, which was recognized for histamine concentrations greater than around 10 mM [33].

Although the DAO-GH showed a high affinity towards the substrate histamine, it showed an even higher affinity towards tyramine, with a *K*_m_ of 0.009 ± 0.0002 mM (V_max_ = 4.3 µkat_Tyramine_/g_Protein_; R^2^ = 0.89). By comparison, other oxidases capable of degrading tyramine showed a distinctively lower affinity, for example, a microbial amine oxidase from *Aspergillus niger* (*K*_m_ = 0.12 mM) [36], a bovine amine oxidase (*K*_m_ = 0.65 mM) [37] and plant amine oxidases from *Lathyrus cicero* (*K*_m_ = 3.1 mM) and *Pisum sativum* (*K*_m_ = 2.3 mM) [37].

The DAO-GH was substrate-inhibited by tyramine (*K*_i_ = 10.4 ± 4.0 mM), which was recognized for tyramine concentrations above approximately 0.5 mM.

Additionally, the DAO-GH activity was determined with the biogenic amines cadaverine and putrescine, which are both aliphatic diamines. The relative DAO-GH activity (compared to the activity with histamine at 1 mM) in concentrations ranging from 0.1 to 5 mM, was merely between 0.1 and 1.3% and 0.1 and 2.3% for cadaverine and putrescine, respectively.

Since the DAO-GH showed the highest activity with histamine and tyramine, but only weak activity with cadaverine and putrescine, the DAO-GH is selective for the most relevant biogenic amines in the food industry. The European Food Safety Authority’s (EFSA) Panel on Biological Hazards stated that “histamine and tyramine are considered as the most toxic and food safety relevant” biogenic amines [12]. Tyramine exerts effects of vasoconstriction in the human body causing symptoms such as hypertension, headache, perspiration, vomiting and pupil dilation [12]. These symptoms are also experienced in the case of histamine intolerance; therefore, the differentiation to a tyramine intolerance might be inconclusive.

### 3.6. Stability of the DAO-GH in SIF

**Figure 6:**
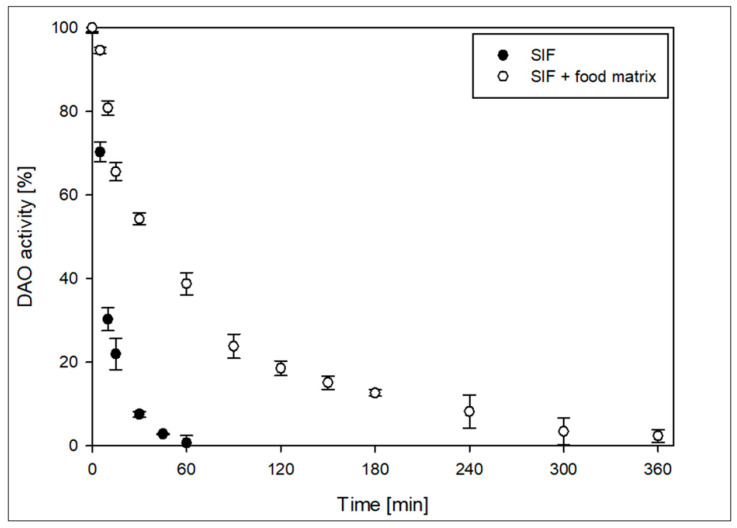
Stability of DAO-GH in simulated intestinal fluid (SIF) and SIF containing established food constituents (BSA, whey protein isolate and sodium caseinate at 16.67 g/L and sucrose at 50 g/L) (SIF + food matrix) at 37 °C. 100% DAO activity (SIF) = 7.7 ± 0.1 nkat/mL. 100% DAO activity (SIF + food matrix) = 5.6 ± 0.1 nkat/mL.

If the DAO-GH is to be used for the degradation of histamine under intestinal conditions, the DAO should be sufficiently stable and active under the conditions prevailing in the human intestine. The United States Pharmacopeia [29] describes a SIF on the basis of monobasic potassium phosphate, sodium hydroxide and with the addition of the enzyme preparation pancreatin. Pancreatin contains a mixture of different digestive enzymes, such as peptidases, amylases and lipases [38]. The partially purified DAO-GH was tested in SIF at 37 °C with and without the addition of known food components to simulate a possible food matrix. (Figure 6).

The DAO-GH had a half-life of around 9 min in pure SIF and no activity after 60 min. The addition of the food matrix increased the half-life of DAO-GH to 47 min. This resulted in the DAO-GH activity being measurable for up to 360 min probably by delaying the proteolytic degradation by providing alternative substrates for the pancreatin peptidases. The relative activity of DAO-GH was 23.8 ± 0.7% after 90 min. In addition, the theoretically available total DAO activity within 90 min is around 50% of the activity applied initially.

### 3.7. Spray Drying of the DAO-GH and Assessment of Its Storage Stability

**Figure 7:**
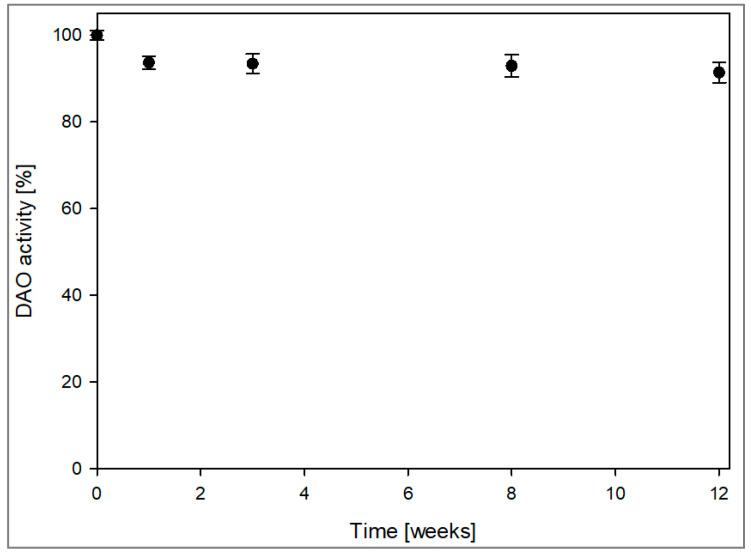
Storage stability of spray-dried DAO-GH at 20 °C. 100% DAO activity = 155 nkat/g_powder_.

The DAO-GH was spray-dried with maltodextrin as the carrier to provide a storable DAO preparation. A total DAO-GH activity of 1970 nkat in a volume of 104 mL (containing 10% (*w*/*v*) maltodextrin) was spray-dried at an outlet temperature of 90 °C with a yield of 83.3%. The specific DAO-GH activity per gram powder was 155 nkat/g_powder_ with a water activity value of 0.226. The DAO-GH powder was stored at 20 °C, protected from sunlight for up to 12 weeks and the DAO activity was determined regularly over this period (Figure 7). After one week of storage, the DAO activity decreased slightly to 93%, but it remained stable at this level over the following weeks. After 12 weeks, the residual DAO activity was still at 93%, demonstrating remarkable stability.

### 3.8. Degradation of Histamine in Tuna Paste

The DAO-GH was applied in a tuna paste for the degradation of histamine. The tuna was spiked with 150 mg/kg histamine before the paste was prepared. The experiment was carried out at a low temperature of 5 °C and pH 5.6. The DAO-GH was added as powder at 0.27% (*w*/*v*) (equal to 40 nkat DAO activity per 100 g tuna). The initial histamine concentration in the paste of 65.8 ± 1.5 mg/L (determined by RP-UHPLC) was reduced by 19.9 ± 2.7% to 52.7 ± 0.3 mg/L after 24 h. The incomplete histamine degradation was explained by the unfavorable experimental conditions (low pH and temperature) and the low amount of DAO added. Since the experimental conditions can be considered as given, future experiments should investigate the use of higher amounts of DAO and a longer incubation period. To date, no DAO preparation has been shown in the literature to degrade histamine under these challenging but realistic conditions. The degradation of histamine in a commercial fish sauce with a DAO from *Natronobeatus ordinarius* was recently investigated by Hou et al., 2024 [18]. Here, 37.9% of the histamine applied initially were degraded within 24 h. The absolute decrease cannot be concluded from the article due to inconclusive data. However, more favorable conditions were chosen since the degradation experiment was performed at 50 °C. The present pH value or whether the pH value of the fish sauce was adjusted were not mentioned in the study.

### 3.9. Degradation of Histamine in SIF

The application of DAO-GH for the degradation of histamine in a SIF in the presence of established food constituents was conducted according to Kettner et al., 2022 [28], but on a 5 mL scale. The DAO-GH powder (2.6 g/L; 2400 nkat/L) was added and it completely degraded the histamine concentration applied initially at 150 mg/L within 90 min at 37 °C. Future experiments must focus on the preparation of DAO-GH tablets as described by Kettner et al., 2022 [28].

## 4. Conclusions

The newly discovered DAO-GH was biotechnologically produced and biochemically characterized in this study. The findings revealed that DAO-GH exhibits biochemical properties that make it suitable for histamine and tyramine degradation both in fermented foods and within the human small intestine. Notably, the DAO-GH demonstrated significant histamine degradation even under challenging conditions, including slightly acidic pH and low temperature. Thus, the newly discovered DAO shows great potential in food production. It could be used as a processing aid to enable the enzymatic degradation of histamine and tyramine. This would allow the production of foods with reduced levels of these biogenic amines, which is particularly relevant for products such as fish, cheese, cured meats and other fermented foods. These foods are often avoided by individuals with sensitivities. Therefore, targeted biogenic amine degradation could pave the way for specific labeling indicating a low histamine and tyramine content. Additionally, the results suggest that DAO-GH could potentially serve as a dietary supplement or therapeutic agent in the treatment of histamine intolerance symptoms. Therefore, the newly discovered DAO-GH holds great potential for diverse applications in the food industry and healthcare. Future experiments must focus on the wide range of applications in different foods and on further improving the biotechnological production of DAO-GH.

## Figures and Tables

**Table 1:** Purification of DAO-GH by polyethyleneimine precipitation (PEI) of nucleic acids, fractionated ammonium sulfate precipitation (AS) and hydrophobic interaction chromatography (HIC). PF = Purification factor.

	Volume [mL]	Protein [g]	DAO Activity [µkat]	Specific DAO Activity [µkat_Histamine_ g_Protein_^−1^]	Yield [%]	PF [-]
**Crude**	410	10.18 ± 0.4	32.9 ± 1.58	3.2 ± 0.16	100	1.0
**PEI**	376	8.43 ± 0.18	31.0 ± 0.33	3.7 ± 0.04	94	1.1
**AS**	670	2.52 ± 0.03	28.6 ± 0.33	11.4 ± 0.13	87	3.5
**HIC**	300	0.80 ± 0.01	15.7 ± 0.39	19.7 ± 0.49	48	6.1

## Data Availability

The original contributions presented in this study are included in the article/Supplementary Materials. Further inquiries can be directed to the corresponding author.

## Supplementary Materials

The following supporting information can be downloaded at: https://www.mdpi.com/article/10.3390/foods14173093/s1, Table S1: Part plasmids used for cassette plasmid construction; Figure S1: Cassette plasmid for the integration of the *dao-gh* gene into the genome of *Komagataella phaffii* ATCC 76273. *Pme*I linearization of the cassette plasmid prior to transformation in *K. phaffii*; Figure S2: (A) Nucleotide and amino acid sequences of the DAO from *Glutamicibacter halophytocola*. The nucleotide sequence was obtained by amplification from the genomic DNA of the *G. halophytocola* isolate and subsequent sequencing. (B): Nucleotide sequence of the DAO from *Glutamicibacter halophytocola*, codon-optimized for the expression in *Komagataella phaffii*; Figure S3: Agarose gel electrophoresis showing the verification of the integration of the DAO-GH expression cassette into the genome of *K. phaffii* by PCR. 1% (*w*/*v*) agarose gel. M = Gene Ruler 1 kb Plus DNA Ladder. WT = *K. phaffii* ATCC 76273. 1–6 = recombinant *K. phaffii* clones. Arrow indicates the expected DNA band for the integration of the expression cassette into the genome of *K. phaffii*. The following primers were used: 5′-gactggttccaattgacaagc-3′ and 5′-ggagcttgcatagaacagtg-3′. PCR was done using TaKaRa Ex Taq DNA Polymerase; Figure S4: Investigation of intracellular, recombinant DAO-GH production in *K. phaffii* using the AOX1 promoter. Cultivation was done in deep well plates in 500 μL working volume at 30 °C using BMGY/BMMY medium. DAO activity was determined after 48 h of cultivation; Figure S5: Chromatogram of the HIC purification of DAO-GH. DAO-GH was eluted in a linear gradient by decreasing the binding buffer and thereby the ammonium sulfate concentration from 1.3 M to zero; Figure S6: Chromatogram of the size-exclusion chromatography (SEC) of DAO-GH (A). Chromatogram of the SEC gel filtration calibration kit (Cytiva, USA) for high molecular weight (B). Calibration curve of the gel filtration calibration kit (C). The SEC was done using the HiLoad 16/600 Superdex 200 prep grade (Cytiva, USA) column on the Äkta pure chromatography system (Cytiva, USA).
