# Supplementary material for: A Diamine Oxidase from Glutamicibacter halophytocola for the Degradation of Histamine and Tyramine in Foods
Source: Foods. 2025 Sep 3;14(17):3093. doi: 10.3390/foods14173093 (PMC12427885; doi:10.3390/foods14173093)
Supplement: Supplementary file 1 [file foods-14-03093-s001.zip › foods-3827263-supplementary.pdf]

A Diamine Oxidase from *Glutamicibacter halophytocola* for the Degradation of Histamine and Tyramine in Foods

Lucas Kettner [1], Alexander Freund [1], Anna Bechtel [1], Judit Costa-Catala [2] and Lutz Fischer [1,*]

[1] Department of Biotechnology and Enzyme Science, Institute of Food Science and Biotechnology, University of Hohenheim, Garbenstr. 25, 70599 Stuttgart, Germany
[2] Departament de Nutrició, Ciències de l'Alimentació i Gastronomia, Campus de l'Alimentació de Torribera, Universitat de Barcelona, Av. Prat de la Riba 171, 08921 Santa Coloma de Gramenet, Spain
* Correspondence: lutz.fischer@uni-hohenheim.de; Tel.: +49-711-459-22311

Supporting information

Table S1: Part plasmids used for cassette plasmid construction.

Plasmid	Type	Description/Parts	Reference
pYTK002	1	ConLS (assembly connector)	(Lee et al., 2015)
pPTK001	2	pAOX1	(Obst et al., 2017)
pPTK-3-DAO-GH	3	codon optimized *dao-gh* gene	This study
pPTK019	4	tAOX1	(Obst et al., 2017)
pYTK072	5	ConRE (assembly connector)	(Lee et al., 2015)
pYTK080	6	ZeocinR	(Lee et al., 2015)
pPTK020	7	*attB* (BxbI recognition site)	(Obst et al., 2017)
pYTK084	8	KanR-ColE1	(Lee et al., 2015)

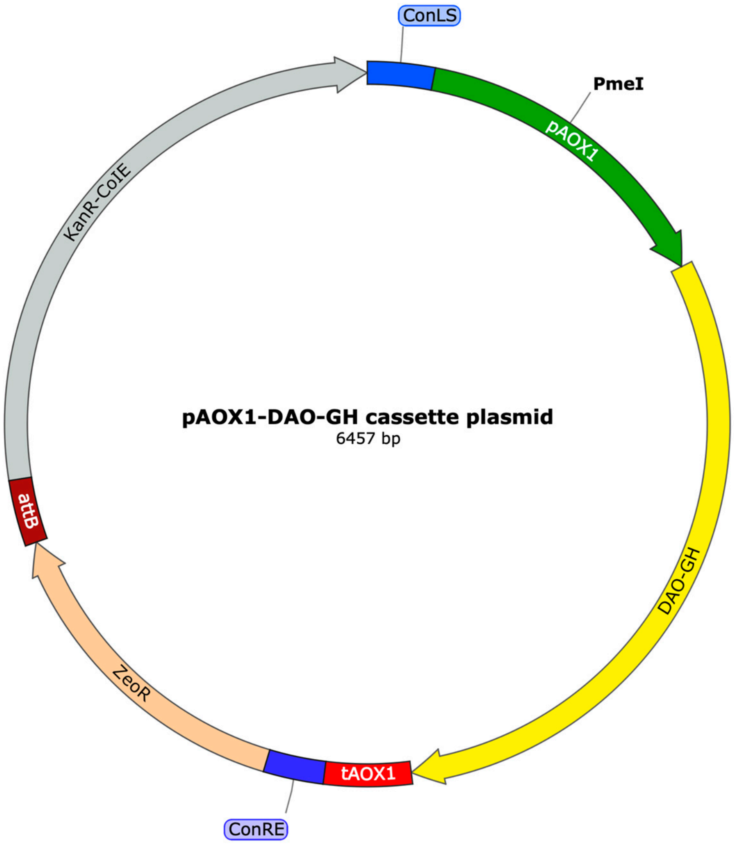

Figure S1: Cassette plasmid for the integration of the *dao-gh* gene into the genome of *Komagataella phaffii* ATCC 76273. *Pme*I linearization of the cassette plasmid prior to transformation in *K. phaffii*

ATGGAACACCTTCACCCAACGACAGCGTTGGAGACCGCGCACCCACTGGAACAGATCACCAGTGAAGAGATCCTGCGTAC
TCGCAAGATCCTTGCCGACGCCGGGCTGGTTGAGCAGACCACCCGTTTCGCCTACCTGGGCTTGCTGGATCCACCCAAGG
ACCTGCTCTATGCCGATGCTGGCACAGAGATTCCGCGCAAGATTCGTGTGATGCTCTACGATCCGACCATCCCCCGCTCC
CTGGACATCACGATCTGCCTGGCCAGCGCCGAGATAGAATCACAGCGCGAAATCGAAGCAGCGACAGAGGGACAAGTTCC
GGTGCTGCTTGAAGAATTCGACACCGTCGAAGAAATCCTGGCCAACGACGAAGGATGGATCAAGGCCTTGGCCAGCCGCG
GACTGAGCACATCCCAGGTTCGAGTCGCACCGCTGTCAGCAGGTGTCTTCGACTACGAGAACGAAGAAGGAAAACGCCTA
TTGCGCGGACTGGGCTTCGTGCAGAATTCCCCTGAGGACCACGCTTGGGCCCACCCCATCGATCGCTTGGTGGCCTTTGT
CGACCTCGAAAACCGTTGCGTTGACCGGCTCATCGATGATGGCCCTGTTCCAGTTCCCGACATCAACGGAAACTACACTGA
TCCACAAGTTCATGGTGAGCTGCGCGATGATCTCAAGGCCATCGAAATCACCCAGCCAGATGGAGCCAGCTTCACCGTGG
ATGGCAACCACCTCTCGTGGCTCGGCTGGGATCTGCGCGTCGGCTTTGACTCACGCGAAGGACTCGTACTCCACCAGATC
CACCACACGCAAGACGGCACGCGTCGCCCGCTGATCCACCGCGCATCCATCTCCGAAATGGTAGTGCCCTACGGCGATC
CGTCCCCTTACCGGAGCTGGCAGAACTATTTCGACACCGGAGAATATTTGGTGGGACGCGACGCGAACTCACTGAAGCTC
GGCTGCGACTGCCTCGGAGAAATCCACTACATGTCTCCCATGGTTGCCGACGATTTTGGCAATCCTCGCGTCATCGACAAC
GGCATCTGCATTCACGAGGAAGACGCCGGAATCGGATGGAAGCACACCGATGAGTGGGCCGGATCCAACGAAGTGCGCC
GCAACCGGCGCCTGGTGGTTTCCTTCTTCACCACCGTGGGCAATTATGACTACGGTTTCTACTGGTACCTGTACCTCGACG
GCACCATCGAGTTTGAAGCCAAGGCCACGGGCATCGTCTTCACCGCGGCGTTGCCGCACAAGGGTTACGAGTACGCCTCA
GAAATCGCGCCGGGCTTGGCTGCGCCATTCCACCAGCATCTCTTTGGCGCACGCCTTGACATGATGATCGACGGGCACGC
CAACGCTGTTGACGAGCTAGAAGTAGTCCGGTTACCGAAGTCCGAAGGCAATCCACACGGCAACGCCTTCACCCAAAGCC
GGCTGCGTTTGGGCACCGAGCAGCAGGCGGTACGCGACGCCAATGCAGCGGCGGGACGCGTATGGCAGGTGAGCAACC
CCGATTCCCTGAATCACGTGGGCGAGCCGGTGGGCTACACGTTGTACCCGCAGAACAATCCGACGCTGGCTATGGCTGAC
GACTCGTCCATCGCTGCCCGTGCCGCCTTTACCCGACACGACCTCTGGGTCACGCGCTTTGCCGAAGGCGAGCTCTATGC
AGCCGGCGACTTCGTCAACCGCAATCCAGGCGGGGCGGGGCTTCCTGCCTTCGTCGAGGCCGACCGGGATATCGACGGA
CAAGATATCGTGTTGTGGCACTCCTTTGGCCTGACCCACTTCCCTCGCCCCGAGGACTGGCCCATCATGCCAGTGGATAC
CGTAGGGTTCACGCTGAAGCCACACGGTTTCTTCAATGAAAACCCGATGCTCAATATCCCTGCCTCGACAAGCTCGCACTG
TTCCATGCAAGCTCCTGAAACCGAAGGACACTGCGGAGCCTAG

MEHLHPTTALETAHPLEQITSEEILRTRKILADAGLVEQTTRFAYLGLLDPPKDLLYADAGTEIPRKIRVMLYDPTIPRSLDITICLASA
EIESQREIEAATEGQVPVLLEEFDTVEEILANDEGWIKALASRGLSTSQVRVAPLSAGVFDYENEEGKRLLRGLGFVQNSPEDHA
WAHPIDRLVAFVDLENRCVDRLIDDGPVPVPDINGNYTDPQVHGELRDDLKAIEITQPDGASFTVDGNHLSWLGWDLRVGFDSR
EGLVLHQIHHTQDGTRRPLIHRASISEMVVPYGDPSPYRSWQNYFDTGEYLVGRDANSLKLGCDCLGEIHYMSPMVADDFGNP
RVIDNGICIHEEDAGIGWKHTDEWAGSNEVRRNRRLVVSFFTTVGNYDYGFYWYLYLDGTIEFEAKATGIVFTAALPHKGYEYAS
EIAPGLAAPFHQHLFGARLDMMIDGHANAVDELEVVRLPKSEGNPHGNAFTQSRLRLGTEQQAVRDANAAAGRVWQVSNPDSL
NHVGEPVGYTLYPQNNPTLAMADDSSIAARAAFTRHDLWVTRFAEGELYAAGDFVNRNPGGAGLPAFVEADRDIDGQDIVLWH
SFGLTHFPRPEDWPIMPVDTVGFTLKPHGFFNENPMLNIPASTSSHCSMQAPETEGHCGA

Figure S2(A): Nucleotide and amino acid sequences of the DAO from *Glutamicibacter halophytocola*. The nucleotide sequence was obtained by amplification from the genomic DNA of the *G. halophytocola* isolate and subsequent sequencing.

ATGGAACACTTGCACCCAACTACTGCTTTGGAAACTGCTCACCCTTTGGAGCAGATTACCTCCGAAGAGATCCTGAGAACC
AGAAAGATTTTGGCTGACGCCGGTTTGGTTGAGCAGACTACTAGATTTGCCTACTTGGGTTTGCTGGACCCACCAAAGGAT
TTGTTGTACGCTGATGCTGGTACTGAGATCCCAAGAAAGATCAGAGTCATGCTGTACGACCCAACTATCCCAAGATCCTTG
GACATTACCATCTGTTTGGCTTCCGCTGAGATTGAGTCCCAGAGAGAAATTGAAGCTGCTACCGAAGGTCAGGTCCCAGTT
TTGTTGGAAGAGTTCGACACCGTGGAAGAGATCTTGGCTAACGACGAAGGTTGGATCAAGGCTTTGGCTTCTAGAGGTTTG
TCCACCTCTCAGGTTAGAGTTGCTCCATTGTCTGCTGGTGTTTTCGACTACGAAAACGAGGAAGGTAAGAGACTGTTGAGA
GGTTTGGGTTTCGTTCAGAACTCTCCAGAAGATCACGCTTGGGCTCACCCAATTGATAGATTGGTTGCCTTCGTCGACTTG
GAGAACAGATGTGTCGACAGATTGATTGACGACGGTCCAGTTCCAGTTCCTGACATCAACGGTAACTACACTGACCCACAA
GTTCACGGTGAGTTGAGAGATGACTTGAAGGCTATCGAGATCACTCAACCAGACGGTGCTTCTTTCACTGTTGACGGTAAC
CACTTGTCTTGGCTTGGTTGGGATTTGAGAGTTGGTTTCGACTCCAGAGAAGGTTTGGTCTTGCACCAGATTCACCACACTC
AAGACGGTACTAGAAGGCCATTGATTCACAGAGCTTCCATTTCCGAGATGGTCGTTCCATACGGTGATCCATCTCCATACA
GATCCTGGCAGAACTACTTCGACACTGGTGAGTACTTGGTTGGTAGAGATGCCAACTCCTTGAAGTTGGGTTGTGACTGTT
TGGGTGAGATCCACTACATGTCCCCAATGGTTGCTGATGACTTCGGTAACCCAAGAGTCATCGACAACGGTATCTGTATCC
ACGAAGAGGACGCTGGTATTGGTTGGAAGCACACTGATGAATGGGCTGGTTCTAACGAGGTCAGAAGAAACAGAAGATTG
GTCGTTTCCTTCTTCACCACCGTTGGTAACTACGACTACGGTTTCTACTGGTACTTGTACCTGGACGGAACTATCGAGTTCG
AGGCTAAGGCTACTGGTATCGTTTTCACTGCTGCTTTGCCACACAAGGGTTACGAATACGCTTCTGAGATTGCTCCAGGTTT
GGCTGCTCCATTTCACCAACATTTGTTCGGTGCCAGACTGGACATGATGATTGATGGTCATGCTAACGCCGTTGACGAGTT
GGAGGTTGTTAGATTGCCAAAGTCTGAGGGTAACCCACACGGTAACGCTTTCACTCAGTCCAGATTGAGATTGGGTACTGA
GCAGCAGGCTGTCAGAGATGCTAATGCTGCTGCTGGTAGAGTTTGGCAGGTTTCTAACCCAGACTCTTTGAACCACGTTGG
TGAGCCAGTTGGTTACACTCTGTACCCACAAAACAACCCTACTTTGGCTATGGCTGACGACTCTTCCATTGCTGCTAGAGCT
GCTTTCACTAGACACGATTTGTGGGTGACTAGATTCGCTGAGGGTGAGTTGTATGCTGCCGGTGATTTCGTTAACAGAAAC
CCAGGTGGTGCTGGTTTGCCAGCTTTTGTTGAAGCTGACAGAGACATTGACGGTCAGGACATCGTTTTGTGGCATTCCTTT
GGTCTGACTCACTTCCCAAGACCTGAGGACTGGCCAATTATGCCAGTTGACACTGTTGGTTTCACCTTGAAGCCACACGGT
TTCTTCAACGAGAACCCAATGTTGAATATCCCAGCCTCCACTTCTTCCCACTGTTCTATGCAAGCTCCAGAAACTGAAGGTC
ACTGTGGTGCTTAA

Figure S2(B): Nucleotide sequence of the DAO from *Glutamicibacter halophytocola,* codon-optimized for the expression in *Komagataella phaffii*.

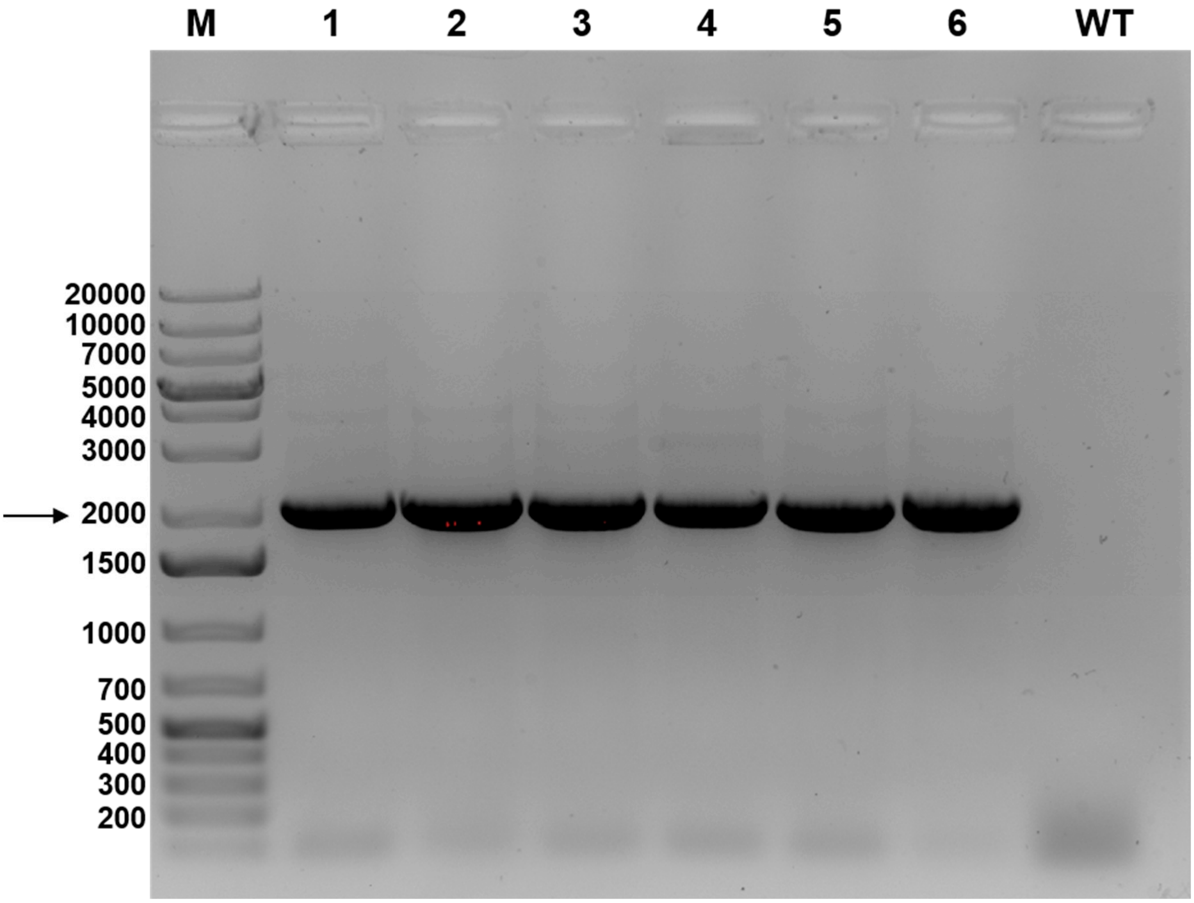

Figure S3: Agarose gel electrophoresis showing the verification of the integration of the DAO-GH expression cassette into the genome of *K. phaffii* by PCR. 1 % (w/v) agarose gel. M = Gene Ruler 1 kb Plus DNA Ladder. WT = *K. phaffii* ATCC 76273. 1–6 = recombinant *K. phaffii* clones. Arrow indicates the expected DNA band for the integration of the expression cassette into the genome of *K. phaffii*. The following primers were used: 5’-gactggttccaattgacaagc-3’ and 5‘-ggagcttgcatagaacagtg-3‘. PCR was done using TaKaRa Ex Taq DNA Polymerase.

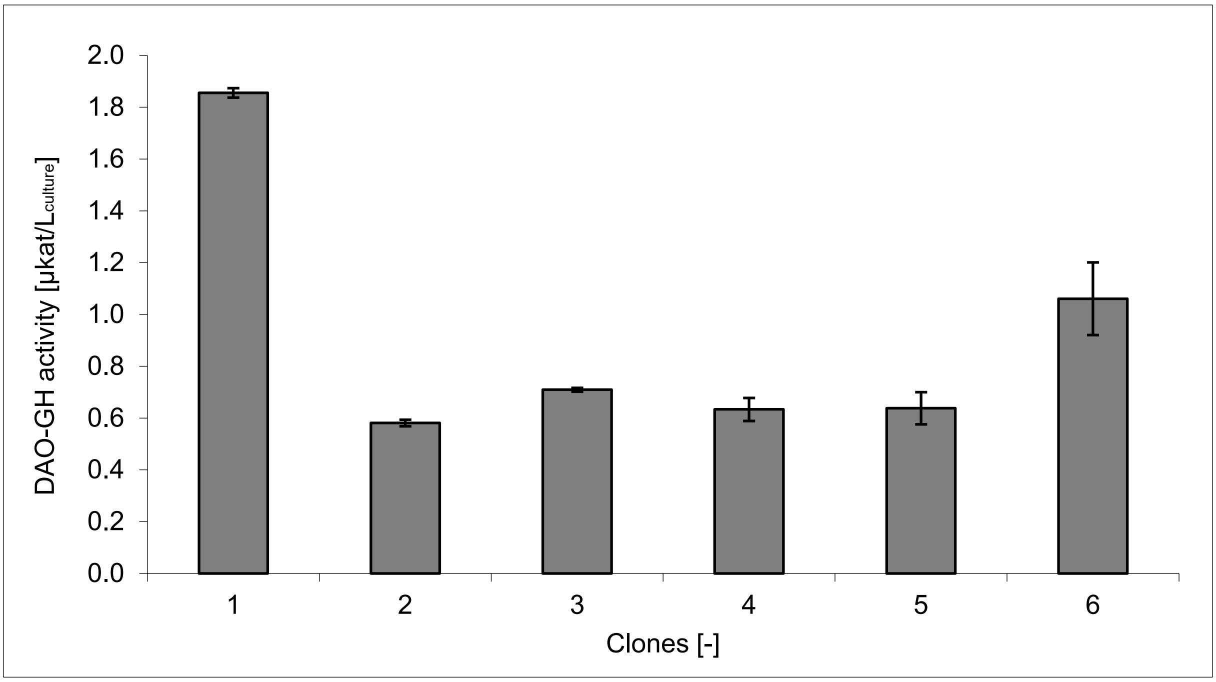

Figure S4: Investigation of intracellular, recombinant DAO-GH production in *K. phaffii* using the AOX1 promoter. Cultivation was done in deep well plates in 500 µL working volume at 30 °C using BMGY/BMMY medium. DAO activity was determined after 48 h of cultivation.

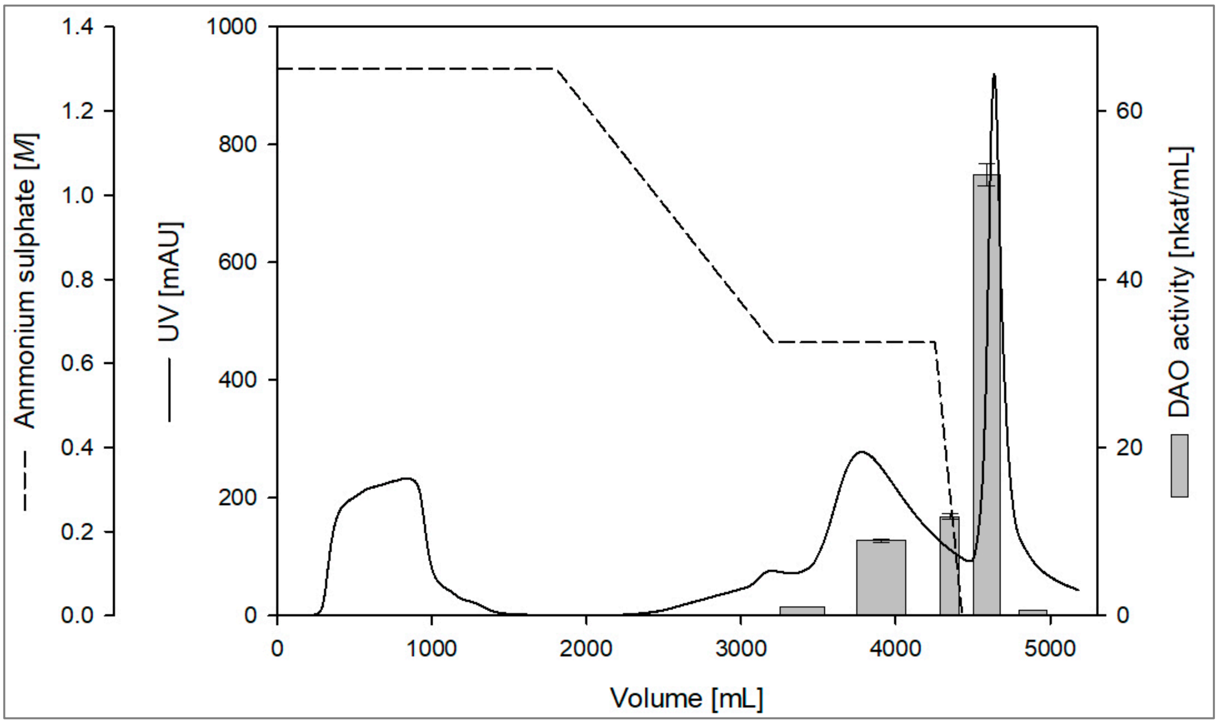

Figure S5: Chromatogram of the HIC purification of DAO-GH. DAO-GH was eluted in a linear gradient by decreasing the binding buffer and thereby the ammonium sulfate concentration from 1.3 M to zero.

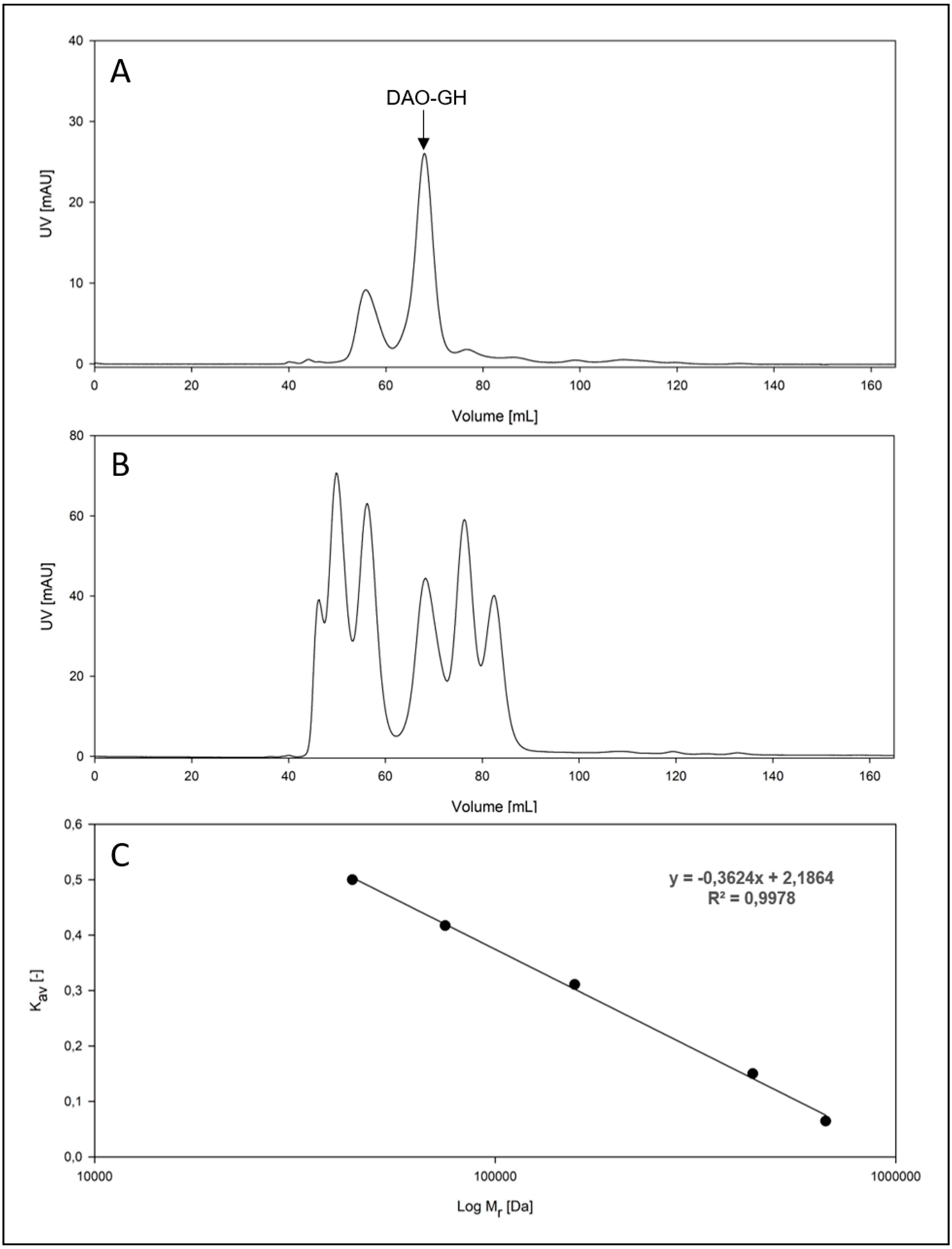

Figure S6: Chromatogram of the size-exclusion chromatography (SEC) of DAO-GH (A). Chromatogram of the SEC gel filtration calibration kit (Cytiva, USA) for high molecular weight (B). Calibration curve of the gel filtration calibration kit (C). The SEC was done using the HiLoad 16/600 Superdex 200 prep grade (Cytiva, USA) column on the Äkta pure chromatography system (Cytiva, USA).

References

Lee, M.E.; DeLoache, W.C.; Cervantes, B.; Dueber, J.E, A highly characterized yeast toolkit for modular, multipart assembly. *ACS Synth Biol* **2015**, *4*, 975–986. https://doi.org/10.1021/sb500366v

Obst, U.; Lu, T.K.; Sieber, V. A modular toolkit for generating *Pichia pastoris* secretion libraries. *ACS Synth Biol* **2017**, *6*, 1016–1025. https://doi.org/10.1021/acssynbio.6b00337
